# Developing target product profiles for *Neisseria gonorrhoeae* diagnostics in the context of antimicrobial resistance: An expert consensus

**DOI:** 10.1371/journal.pone.0237424

**Published:** 2020-09-01

**Authors:** Cecilia Ferreyra, Jennifer Osborn, Francis Moussy, Emilie Alirol, Monica Lahra, David Whiley, William Shafer, Magnus Unemo, Jeffrey Klausner, Cassandra Kelly Cirino, Teodora Wi

**Affiliations:** 1 Foundation for Innovative New Diagnostics (FIND), Geneva, Switzerland; 2 World Health Organization (WHO), Geneva, Switzerland; 3 Global Antibiotic R&D Partnership (GARDP), Geneva, Switzerland; 4 WHO Collaborating Centre for Sexually Transmitted Infections and Antimicrobial Resistance, New South Wales Health Pathology, Microbiology, The Prince of Wales Hospital, Randwick, Australia; 5 Faculty of Medicine, The University of New South Wales, Sydney, New South Wales, Australia; 6 Centre for Clinical Research, The University of Queensland, Brisbane, Queensland, Australia; 7 Department of Microbiology and Immunology, Emory University School of Medicine, Atlanta, Georgia, USA and Veterans Affairs Medical Center, Decatur, Georgia, United States of America; 8 WHO Collaborating Centre for Gonorrhoea and Other STIs, National Reference Laboratory for STIs, Örebro University Hospital, Örebro, Sweden; 9 Division of Infectious Diseases, University of California and David Geffen School of Medicine Los Angeles, Los Angeles, CA, United States of America; McGill University Health Centre, CANADA

## Abstract

**Background:**

There is a need for a rapid diagnostic point of care test to detect *Neisseria gonorrhoeae* (NG) infection to prevent incorrect, lack or excess of treatment resulting from current syndromic management in low-resource settings. An assay to identify NG antimicrobial resistance (AMR) is also highly desirable to facilitate antibiotic stewardship. Here we describe the development of two target product profiles (TPPs): one for a test for etiological diagnosis of NG and *Chlamydia trachomatis* (CT) (TPP1) and one for the detection of NG AMR/susceptibility (TPP2).

**Methods:**

Draft TPPs were initially developed based on a landscape analysis of existing diagnostics and expert input. TPPs were refined via an online Delphi survey with two rounds of input from 68 respondents. TPP characteristics on which <75% of non-industry respondents agreed were further discussed and revised by an expert working group.

**Results:**

The need for a test to identify NG in patients with urethral or vaginal discharge was identified as a minimal requirement of TPP1, with a test that can diagnose NG in asymptomatic patients as the optimal requirement. A sensitivity of 80% was considered acceptable, either in context of syndromic management or screening high-risk populations. For TPP2, the agreed minimal requirement was for a test to be used at level 2 healthcare facilities and above, with an optimal requirement of level 1 or above. A lateral flow format was preferred for TPP1, while it was considered likely that TPP2 would require a molecular format. A total of 31 test characteristics were included in TPP1 and 27 in TPP2.

**Conclusions:**

Following the working group revisions, TPPs were posted online for public feedback for two months, and are now finalized. The final TPPs are currently guiding the development of new diagnostics that meet the defined characteristics to reach the market within two years.

## Introduction

According to the World Health Organization (WHO) in 2016, there were an estimated 376 million new infections globally of the four curable sexually transmitted infections (STIs)–*Chlamydia trachomatis* (CT), *Neisseria gonorrhoeae* (NG), *Treponema pallidum* subspecies *pallidum* and *Trichomonas vaginalis*. Gonorrhoea, caused by the bacterium *Neisseria gonorrhoeae* (NG), is the second most common bacterial STI with an estimated yearly burden of 87 million cases worldwide [1, 2]. Over time NG has progressively developed resistance to the majority of available antibiotics, raising concerns for the longevity of remaining and future therapies [3–5]. There are currently only two new antibiotics in development–zoliflodacin and gepotidacin—and, should either of them be successfully registered, their future conservation will be of primary importance [6, 7]. The WHO syndromic approach for the management of patients presenting with symptoms or signs of STIs has been in use since the 1990s in most resource-constrained settings due to the lack of affordable and appropriate diagnostic solutions [8]. However, its low sensitivity and specificity, and its lack of application for asymptomatic patients and women in general, drives both unnecessary use of antibiotics, and inadequate diagnosis and treatment [9–11]. In order to ensure that current and new antibiotics are used in a responsible manner and to prevent incorrect, lack of or excess of treatment, it is essential to improve and broaden diagnostic capabilities. A rapid point of care test (POCT) to detect NG would be a critical first step to avoid unnecessarily treating patients with STI syndrome, whilst a second assay to identify NG resistance/ susceptibility to existing antibiotics is highly desirable to optimize therapy and facilitate stewardship of both existing and future therapeutic options. To meet patient and market needs, the requirements of such assays should be specified in target product profiles (TPPs).

### Gonorrhoea, a priority pathogen in the era of antimicrobial resistance (AMR)

Gonorrhoea affects high, middle and low-income countries with the African region having the highest incidence rates of infections worldwide, with approximately 50 and 100 estimated new infections per 1,000 women and men, respectively, every year [1, 12]. Urogenital gonorrhoea may be asymptomatic in 40% of men [13] and manifest most commonly as urethritis while it is also asymptomatic in more than half of the women, which increases the risk of misdiagnosis [14]. Women in resource-constrained settings are disproportionately affected by STIs, in particular by serious reproductive tract complications and sequelae [15, 16], such as infertility, pelvic inflammatory disease, ectopic pregnancy, spontaneous abortion, premature delivery and low birthweight. Furthermore, gonorrhoea enhances the transmission and acquisition of HIV infection [17]. Gay, bisexual and other men who have sex with men (MSM), racial or ethnic minorities, indigenous populations and sex workers appear to bear disproportionate burdens of NG. Asymptomatic, particularly extra-genital, gonorrhoea is significantly common in men who have sex with men (MSM) which remains undiagnosed and untreated and may lead to a reservoir which can result in widespread transmission among multiple partners [18, 19].

Appropriate STI diagnosis and treatment is crucial to prevent transmission and sequelae of NG infections [20]; however, access to diagnostic tools to be used at level 1; defined as a health post and centres where simple diagnostic technics, including collection of dried blood sports and rapid or dipstick test can be performed; is scarce in most low- and middle-income countries (LMICs), resulting in misdiagnosis and/or unnecessary antibiotic treatment [21–24]. NG identification can be done by Gram stained microscopy, although it cannot distinguish from other Neisseria species; culture, or the current gold standard, a nucleic acid amplification test (NAAT) [25–29]. A number of NAAT assays are available, however these platforms require significant infrastructure, resources and equipment, including continuous power, clean running water and climate control [27]. In order to reach patients in resource-limited settings, patient samples must be transported from urban, peri-urban and rural settings to the laboratory for processing. This is done using sample transport networks in-country; but, frequently, these services are not well developed, leading to long delays in returning sample results to patients and loss to follow-up [26, 27, 30].

A landscape analysis for STI POCTs performed by WHO showed that there were a limited number of immunoassays designed for use at the point of care available, four of which have been evaluated: the ACON Duo [31], NG ACON Plate (ACON Laboratories, USA) [31], BioStar Optical ImmunoAssay-Gonorrhea (BioStar, Inc., USA) [32], and the One Step Gonorrhea RapiCard InstaTest (Cortez Diagnostics, USA) [33]. The conclusion of this analysis was that although current lateral flow assays (LFA) rapid diagnostic test (RDT) for NG often have specificities of >90%; the sensitivities are often 50% or lower, and as such, do not perform adequately enough to be used for NG diagnosis. Accordingly, improved assays are required; this need is particularly acute with respect to women, where the syndromic approach to managing STIs is inadequate [34, 35]. There are several molecular platforms available and under development for the rapid detection of NG at level 1, some of them include the detection of resistance to ciprofloxacin but still there is no molecular platform able to detect resistance to broad spectrum cephalosporin, and some of them could potentially close the diagnostic gap in LMICS in the future [28, 35–37].

NG has a remarkable ability to develop or acquire resistance to antibiotics, and in the absence of a vaccine antibiotics are almost exclusively relied upon globally for gonococcal disease management and control. NG can very efficiently develop or acquire AMR through mutations, and via horizontal gene transfer (transformation), especially with the non-gonococcal *Neisseria* species of the pharynx. NG has developed resistance to the majority of available classes of therapeutic antibiotics including sulphonamides, penicillins, early-generation cephalosporins, macrolides, tetracyclines and fluoroquinolones, which cannot now be used in most areas of the world [5, 38–40]. Decreased susceptibility or resistance in NG to extended-spectrum cephalosporins, the last remaining option for first line empirical monotherapy, has been reported worldwide [41–43]. Determining whether antibiotic therapy will be effective ideally requires detection of AMR. This therefore poses the extraordinary challenge of developing a rapid diagnostic tool for NG that is also able to identify most AMR determinants [44].

The WHO, the Foundation for Innovative New Diagnostics (FIND) and the Global Antibiotic Research & Development Partnership (GARDP) have been collaborating to ensure antibiotic stewardship of existing and new antibiotics for NG since 2018. The need for more sensitive, easier-to-use and cheaper tests for both CT and NG that can deliver results in a single patient visit was the main rationale for the definition of the TPPs for diagnostics.

Two TPPs have been developed for NG POCT in resource-constrained settings; the first (TPP1) for a test for etiological diagnosis of NG and CT, and the second (TPP2) for the detection of NG AMR determinants that can predict resistance/susceptibility to existing and upcoming antibiotics. Novel technologies for NG detection and AMR prediction are being developed and it is important to ensure they meet the needs of end users particularly in resource-constrained settings [36, 45, 46].

Herein, we describe the TPP development process, which involved a meeting of sexual health, microbiology, diagnostic and global health experts, and the final consensus characteristics for tests to identify NG and CT and the susceptibility or resistance profile of NG to currently used antibiotics.

## Methodology

The purpose of a TPP is to inform product developers of the technological performance standards and operational characteristics of a diagnostic test that are required to meet the end users’ needs for a defined use case [41]. A TPP must be sufficiently detailed to guide developers but not so restrictive as to hamper development.

**Fig 1:**
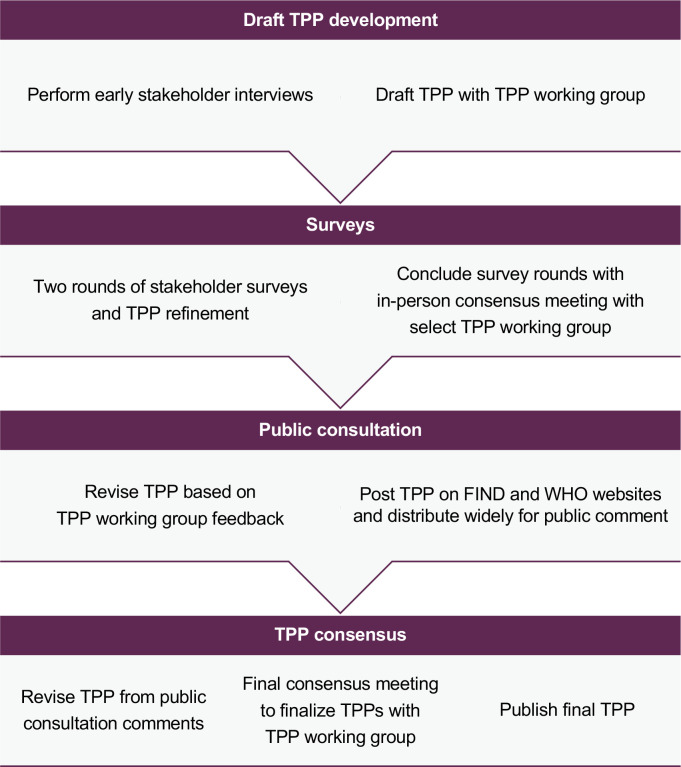
Overview of TPP development process.

A landscape review of NG and/or NG/CT diagnostics available or in the pipeline was the initial step to define the TPP. An expert meeting was then jointly organized by GARDP, FIND and WHO in Geneva, Switzerland in May 2018 and involved key experts to identify the main test characteristics to inform draft TPPs. To further refine this draft, several interviews with key experts were conducted and the draft TPPs were further revised with a technical working group. These drafts were then included in an online Delphi survey process that involved two serial survey rounds of input from 68 survey respondents. Under the Delphi process, experts were asked to rate each TPP characteristic using a Likert scale of 1–5 to indicate level of agreement. The results were analysed with a pre-specified consensus threshold at >50% and >75% agreement (corresponding to a Likert score of either 4 or 5). Industry and non-industry responses were analysed separately; industry responses were only considered advisory and were not included in the main results. In total, 61 non-industry participants (primarily researchers, medical doctors, clinical officers and laboratory experts) and 7 industry participants responded to the Delphi survey rounds. Fig 1.

The survey feedback was presented at a second expert meeting held in March 2019 in Montreux, Switzerland. The TPP requirements that had less than 75% agreement in the survey were discussed, proposed revisions were drafted in consultation with experts and then voted upon to confirm agreement. Any participants with conflicts of interest due to receipt of research funding, advisory fees or supplies from the diagnostic industry, or involvement in the development of diagnostic tests for NG, were excluded from voting. Final TPPs are published at websites of both FIND and WHO:

https://www.finddx.org/wp-content/uploads/2019/09/NG_CT-Test-TPP_20190731_clean-who.pdf

https://www.finddx.org/wp-content/uploads/2019/09/Comprehensive-NG-test-TPP_20190731_clean-who.pdf

## Results

### TPP1 (NG/CT diagnostic test): Scope of the test

The working group identified the need for a test to identify NG in patients with urethral or vaginal discharge as a “minimal requirement” as most patients with symptoms are being managed with WHO syndromic approach and are frequently over-treated with several broad-spectrum antibiotics. The aim of such a test would be to ensure antibiotic stewardship providing NG treatment only to all who test NG positive, and the NG negative patients will likely be likely treated based on syndromic management. This test should be rapid, easy to use, and affordable, have acceptable sensitivity and specificity, be possible to implement at the primary health care (PHC) level in LMICs, and meet the ASSURED criteria [47].

The need for a test that could be used to diagnose NG in asymptomatic patients was highlighted and stated in the TPP as “optimal requirement”, acknowledging that such a test would be technically challenging because of the higher sensitivity required [34, 33]. Asymptomatic patients are the population driving NG transmission [46, 48, 49], hence screening was assigned as an optimal requirement particularly in the context of pre-exposure prophylaxis (PrEP) for HIV infection and antenatal care clinics (ANC).

As the WHO goal is to switch from syndromic management to etiologic diagnosis of STIs [50], it was proposed that the minimum requirement could be to assist in this shift and manage patient care and antibiotic usage more rationally, with an optimal requirement for additional use in etiologic screening to recognize undiagnosed cases and reduce the overall transmission of infection.

It was noted that the TPP should specify that the test can be for NG or NG and CT, to avoid implying that a CT-only test is acceptable. Furthermore, as it may take separate efforts to develop NG and CT tests, and performance characteristics will probably be different across the two, it would be advisable to allow for the option of an NG-only test.

The TPP working group agreed that 80% sensitivity would be acceptable, either in the context of syndromic management or in the context of screening of high-risk populations that cannot be screened using NAAT, as in the latter, there is no risk of overtreatment. Additionally, reaching 90% sensitivity using existing antibodies may be a challenge; while advancements in technology will allow some optimization, it is unlikely that they are sufficient to reach sensitivities of >90% while maintaining the rapidness, ease of use and low cost of the POCT. The appropriateness of implementing a test with lower sensitivity for use in LMICs has always been debated but has been shown to be more cost effective than a syndromic management approach lacking testing. Therefore, the experts agreed a sensitivity of 80% was the lowest acceptable sensitivity while still demonstrating benefit beyond syndromic management without diagnostic test [51–53]. The impact of sensitivity on uptake needs to be considered in conjunction with time, cost and ease of use. For example, a ‘pregnancy test’ style RDT would have greater uptake than a more complex and expensive test with the same sensitivity that takes 30 minutes to return a result [54–56]. The group agreed to break out the sensitivity and specificity in the TPP by test type to allow different values for molecular and non-molecular tests. It was agreed that a molecular test should not go below 90% sensitivity whereas a lateral flow assay could have a lower sensitivity and still be a significant improvement over syndromic management, particularly for symptomatic patients and women in general. The minimum and optimal sensitivity requirements for a non-molecular test could be the same, as it will be specificity that needs to differ.

WHO has developed simplified flowcharts to guide implementation of the syndromic management [57], which is based on the identification of symptoms and signs and the provision of treatment to deal with most of the organisms responsible for producing STIs. A recent systematic review and modelling study on vaginal discharge, performed to inform the TPP development, showed that the pooled sensitivity and specificity of the WHO flowchart 1, consisting of the basic clinical procedures such as history taking including risk assessment, physical examination and bimanual palpation which allows for immediate treatment of CT and NG, is 27.9% (24.7 to 31.1) and 57.0% (56.1 to 58.0) respectively [58]. The absolute effect of differences in prevalence using the pooled sensitivities and specificities of the different vaginal discharge flowcharts reveal that the low diagnostic accuracy of vaginal syndromic case management results in a high number of false positives (lower specificity), leading to overtreatment. The absolute effects on outcomes in settings with different CT/NG prevalence using hypothetical RDTs with sensitivities of 60%, 70%, 80% and a specificity of 90%, and using a POCT molecular assay (sensitivity of 95% and specificity of 98%), revealed fewer false positives and false negatives and more true positives compared with syndromic case management.

**Fig 2:**
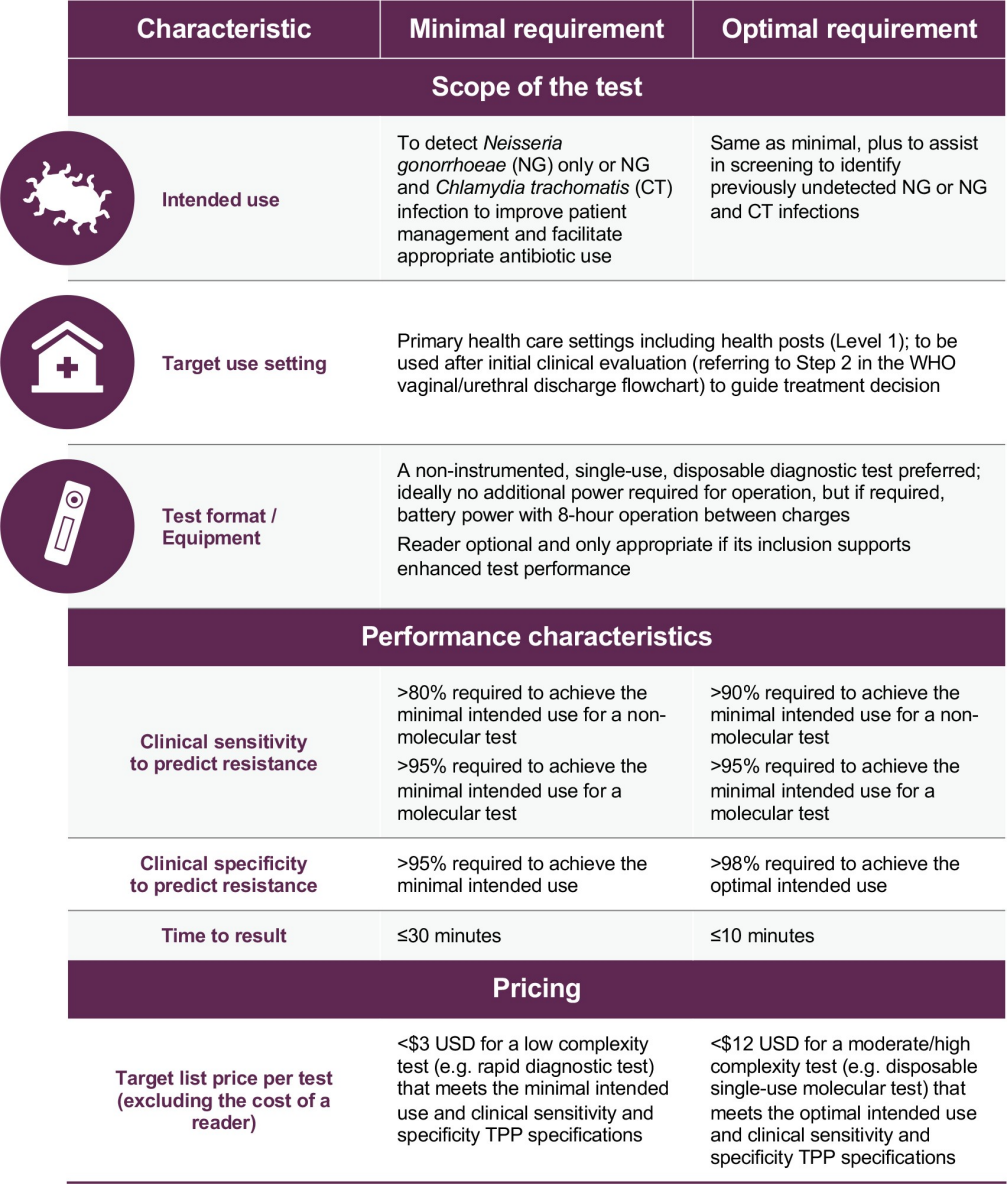
TPP1 for a rapid, low cost diagnostic to identify gonorrhoea with or without chlamydia infection.

A total of 31 test characteristics were included in TPP1. Summary of TPP 1 is showed in Fig 2.

### TPP2 (AMR test): Scope of the test

In the context of AMR, the expert group agreed on the need for a rapid test that allows resistance/susceptibility testing and individualized treatment for patients with confirmed NG, but also the need for increased AMR surveillance in LMICs, particularly in Africa, Eastern Europe, Central America, the Caribbean, and Asia, where substantially improved NG AMR data are urgently needed. However, the TPP2 used in the Delphi survey focused on patient management. The test was intended as a reflex test to be used after confirming NG infection in order to ensure antibiotic stewardship, as a minimum requirement. Individualized treatment is a more feasible use case but this is only likely to be possible at level 2 of the health care system–defined as a district hospital, which can act as a referral centre for specimens sent from level 1, include dedicated laboratory space, trained technicians and reagents) if an instrument is required for detection of antimicrobial resistance/susceptibility [22].

It was assumed that a test based on genotypic markers of infection would likely require a molecular testing format. For level 1 use [22], the most viable option would be a non-instrumented approach, such as a single use disposable LFA. However, if an instrument is required, it would be preferable for the instrument to have the capacity for testing of other diseases that commonly present at this level of care, which can be challenging from a cost perspective.

A test that could predict AMR/susceptibility to currently available antibiotics (particularly ceftriaxone and cefixime) and new antibiotics under development for NG would be ideal, however the technical challenges associated with developing such an assay mean that other key antibiotics might need to be chosen for inclusion in the assay. Several molecular tests under development include the detection of ciprofloxacin resistance/susceptibility [59, 60]; however, its utility in most African and South East Asian regions would be limited due to the high levels of resistance to this antibiotic. Whilst there is potential to develop a penicillin high level resistance test based on beta-lactamase production, the experts were concerned about how to deal with lower level or intermediate non-beta-lactamase (chromosomal) resistance to penicillin, as the predictive value of relevant markers is not fully understood, and treating people with intermediate resistance will likely drive more resistance.

Development of cephalosporin resistance tests would be extremely challenging but are also the most needed. Detection of mosaic *penA* alleles may have low specificity for predicting resistance (so as to rule out use of a cephalosporin) as susceptible strains can also carry most of the mosaic *penA* alleles. Additionally, in some countries such as China, Korea and Vietnam, much of the cephalosporin resistance reported has been due to mutations in non-mosaic alleles [5, 42, 61], therefore impacting upon the sensitivity of a mosaic *penA* based test. The possibility of including testing for novel antimicrobials, such as zoliflodacin, gepotidacin or other, was discussed, but it was noted that the knowledge relating to mechanisms of clinical resistance was limited for these drugs, and that whilst selecting for resistance in the laboratory is possible, the mechanism selected for will not necessarily be the dominant mechanism in a real-world situation once the antibiotic is in use.

**Fig 3:**
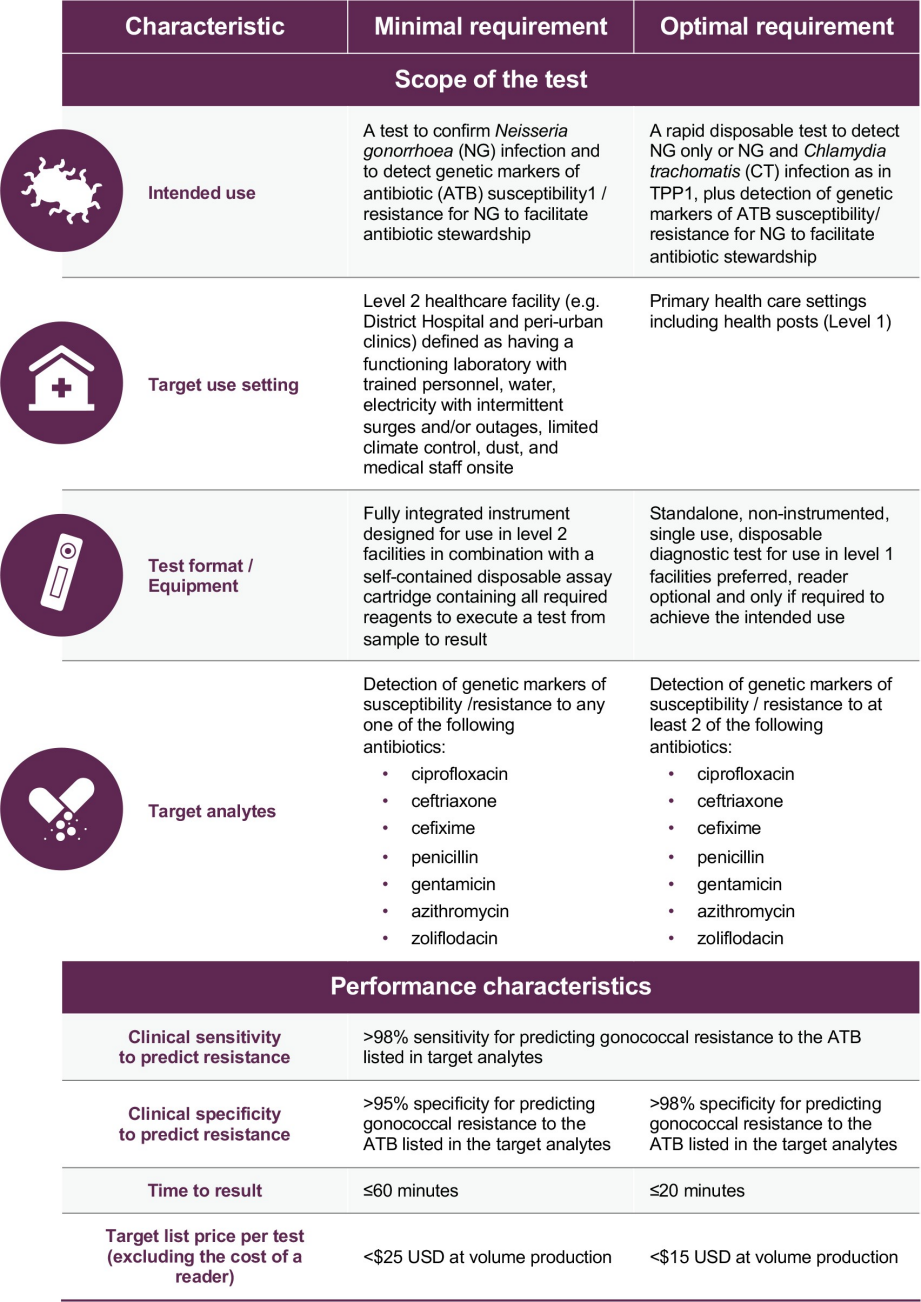
TPP2 for a test to identify susceptibility/resistance of gonorrhoea to antibiotics to facilitate antibiotic stewardship.

Given the difficulties in developing AMR tests for many existing antibiotics, it was agreed to focus on tests for a single antibiotic for the minimum requirement, and a test including more than one antibiotic for the optimal requirement. This way, all of the antibiotics of interest could be listed, leaving options open for developers. A total of 27 test characteristics were included in TPP2. Summary of TPP 2 is showed in Fig 3. The final/revised TPP is shown in S2 File.

## Discussion

Defining and disseminating TPPs for diagnostic tests identified as high priority helps to guide product developers. Consultation of a broad range of experts, and clarity of process, is required to make informed decisions about product features and specific performance specifications. The Delphi process and expert consultations used in the development of the NG TPPs provides confidence in the robustness of the final versions. This expert approach to define assay requirements should guide product development and enable targeted and timely efforts by industry partners and academic institutions.

It was agreed that the most suitable type of test for TPP1 for LMICs would be a LFA format due to the ease of use and low cost; although the few tests commercialized in the past showed poor performance and were abandoned years ago, several molecular assays are now in the pipeline [33, 35, 62]. The WHO syndromic approach has shown limitations in ensuring that patients with STIs are accurately diagnosed and treated, but most LMICs would face challenges in paying for a test that would be more expensive than the current combination of ceftriaxone and azithromycin (1 USD dollar) [63, 64, 65]. Patient health seeking behaviour and country guidelines for STI treatment are recognized as a major barrier for the implementation of such tests. Only half of patients with gonorrhoea have symptoms and only a third of them will present for health care [54, 66], meaning that many cases are missed from the health system, which would therefore benefit from a test with higher sensitivity that can be effectively used for screening as specified in the optimal requirements for TPP1.

Regarding TPP2, it was agreed that the minimum requirement should be for a test to be used at district hospital level and above, with an optimal requirement of use at PHC level or above; however, it will be important to specify that the minimum requirement test will not change patient management at PHC level. It was noted that if sample is sent to district hospital for testing, but the patient is lost to follow up, the test becomes a surveillance tool rather than a tool for patient management. If this is the intended use case, the TPP could be less prescriptive, as there is the potential to use a more complex sequencing-based system for surveillance purposes. Selecting the appropriate AMR determinants to be included in the test is a major challenge due to the genetic adaptability of NG allowing it to quickly develop or acquire AMR determinants; additionally, manufacturers might need to update the test as novel AMR mechanisms develop, which implies a need to resubmit the updated product for regulatory approval. However, this may be an opportunity for regulatory authorities to provide a more flexible framework, given the public health implications of being able to change the AMR determinants detected in a diagnostic assay.

The type of instrument and price were identified as some of the most important barriers for implementation of both TPPs. Most LMICs have a set of common barriers to implementation of diagnostic tests: a) deficient laboratory structures; b) lack of dependable sources of electricity and cold chain; and c) lack of trained staff [67]. Increased access to these diagnostic products will require the development and deployment of an instrument that can be used in remote LMICs by persons with little or no laboratory training. It was agreed that TPP1 should be ideally instrument free but given the technical challenges to develop such a tool, a small portable and battery-operated platform would be acceptable. TPP2 will require a higher level of infrastructure, which could be a district or regional hospital; however, this will not ensure individualized patient management if samples need to be referred. The experts acknowledged that the cost calculation would be different for both instruments, making TPP2 more expensive. A successful AMR test will be paradigm-shifting and likely to be included in a global AMR strategy rather than being restricted to LMICs, thus a strategy of income-based differential pricing might be possible. Additionally, fewer tests would be required as only those who have tested positive for NG would receive the AMR test, reducing overall funding needs.

Accurate diagnosis of STIs, and in particular for NG, is critical to ensure appropriate treatment, and for antibiotic stewardship to reduce the burden of AMR in the near future. However, structural challenges remain associated with the implementation of such tools once they are developed. Country adoption of new assays, and willingness to pay, are amongst the key barriers identified in a recent market assessment performed by FIND (manuscript under preparation). Acceptance of a new diagnostic approach will involve a cultural change from syndromic management in many LMICs. STI programmes are not sufficiently supported by donors in these settings, and even though some countries make efforts to integrate STIs into HIV programmes, integration challenges have been identified, including a lack of policy and guidelines, inadequately trained providers, vertical programming, provider work overload, and a weak health system [68].

As a next step these TPPs will serve to support the development of new diagnostics that meet the defined characteristics. It is anticipated that such tools may become available in the following two years.

## Supporting information

S1 File (PDF)

S2 File (PDF)
