# Supplementary material for: Developing target product profiles for Neisseria gonorrhoeae diagnostics in the context of antimicrobial resistance: An expert consensus
Source: PLoS One. 2020 Sep 1;15(9):e0237424. doi: 10.1371/journal.pone.0237424 (PMC7462286; doi:10.1371/journal.pone.0237424)
Supplement: S1 File — (PDF) [file pone.0237424.s001.pdf]

Target product profile
for a rapid, low-cost diagnostic to distinguish gonorrhoea from Chlamydia infection at primary care

FIND, Campus Biotech. Chemin des Mines 9, 1202 Geneva, Switzerland
T +41 (0)22 710 05 90, F +41 (0)22 710 05 99, www.finddx.org

Target product profile for a rapid, low-cost diagnostic to distinguish gonorrhoea from Chlamydia infection at primary care		
**Characteristic**	**Minimal**	**Optimal**
**SCOPE**		
**1. Intended use**	To detect Neisseria gonorrhoea (NG) only or NG and Chlamydia trachomatis (CT) infection to improve syndromic patient management and to facilitate appropriate antibiotic use	Same as minimum plus to assist in screening to identify previously undetected NG or NG and CT infections to support public health management
**2. Target use setting**	Primary health care settings including health posts (Level 1[1]); to be used after initial clinical evaluation (referring to Step 2 in the WHO Vaginal/Urethral Discharge Flowchart[2]) to guide treatment decision	
**3. Test format / Equipment**	A non-instrumented, single use, disposable diagnostic test preferred; Ideally no additional power required for operation, but if required, battery power with 8-hour operation between charges.<br>Reader optional and only appropriate if its inclusion supports enhanced test performance (see Appendix 1 for reader requirements)	
**4. Target users**	The target users include community health workers with minimal training and any health worker with a similar or superior training level	
**5. Target analytes**	Identification of NG or NG and CT	Same as minimal, plus detection additional sexually transmitted infections (e.g. Mycoplasma and trichomonas) ideal
**PERFORMANCE CHARACTERISTICS**		

[1] Ghani AC, Burgess DH, Reynolds A, Rousseau C (2015). Expanding the role of diagnostic and prognostic tools for infectious diseases in resource-poor settings. *Nature* 528: S50-52

[2] https://apps.who.int/iris/bitstream/handle/10665/43275/9241593407_mod4_eng.pdf?sequence=5&isAllowed=y

**6. Clinical Sensitivity[3]**	>80% required to achieve the minimal intended use for a non-molecular test;<br>>90% required to achieve the minimal intended use for a molecular test	>90% required to achieve the minimal intended use for a non-molecular test;<br>>95% required to achieve the minimal intended use for a molecular test
**7. Clinical Specificity[1]**	>95% required to achieve the minimal intended use	>98% required to achieve the optimal intended use
**8. Specimen[4]**	Women: self-collected and provider-collected high vaginal swabs<br>Men: urethral swab acceptable, urine preferred<br>Same test format able to accept multiple specimen types to achieve results for men and women	Women: urine preferred, self-collected and provider-collected high vaginal swabs acceptable<br>Men: urine, and rectal and pharyngeal swabs<br>Same test format able to accept multiple specimen types to achieve results for men and women
**9. Analytical Inclusivity**	Assay detects geographically and genetically representative Neisseria gonorrhoea strains	
**10. Analytical Exclusivity**	No cross-reactivity with >95% of non-chlamydial and non-gonococcal pathogens and other microorganisms that frequently colonize and/or infect the genital tract especially non-gonorrhoeal Neisseria	No cross-reactivity with >99% of non-chlamydial and non-gonococcal pathogens and other microorganisms that frequently colonize and/or infect the genital tract especially non-gonorrhoeal Neisseria
**OPERATIONAL CHARACTERISTICS**		
**11. Specimen preparation**	Minimal sample processing; no more than one operator step	Integrated; no sample preparation required by user
**12. Steps performed by healthcare worker between specimen preparation and result**	No more than three operator steps, none of which is timed or labour intensive	One operator step (none of which has a timed interval), excluding waste disposal
**13. Additional consumables required but not provided within the test kit**	None, except for specimen collection	

[3] In genital specimens with performance verified as compared to nucleic acid reference standard

[4] Sensitivity and specificity for rectal and pharyngeal swabs is not yet determined

**14. Cold chain**	None required at any point	
**15. Test kit**	All materials required for test procedure, including devices, reagents or other consumables to diagnose one individual, included in packaged, self-contained kit (either packaged individually as one test per test kit or sufficient to perform the number of tests packaged in the test kit box – e.g. 20, 50 or 100 tests)	
**16. Test kit stability and storage conditions**	12 months, stable between 2-35°C, 70% humidity, 3000 meters altitude	18 months, stable between 0-50°C, 90% humidity, 3000 meters altitude
**17. Environmental tolerance of packaged test kit**	• Transport stress (48 hours with fluctuations up to 50 °C and down to 0 °C)<br>• Tolerate exposures between 2 °C and 45 °C at an altitude up to 3000 meters, up to and including condensing humidity	
**18. Operating conditions**	• Operation between 15 °C and 40 °C at an altitude up to 2000 meters<br>• Extremely low relative humidity to condensing humidity<br>• Result interpretation in low light settings	Same, plus operation between 10 °C and 45 °C at an altitude up to 3000 meters preferred
**19. Training required**	< 90 minutes	30 minutes
**20. Clean water**	None required	
**21. Time to result**	≤30 minutes	≤10 minutes
**22. Duration of sample stability (time from specimen collection to insertion into test cartridge)**	Immediate testing of the sample	
**23. Stability of valid result (read window)**	At least 30 minutes (after which results may be *false* or *invalid*);<br>Clear language in the instructions for use regarding test reading	≥1 hour (after which results give *invalid* rather than *false* results);<br>Clear language in the instructions for use regarding test reading
**24. Safety precautions (bio-safety requirements)**	Closed, self-contained system; unprocessed sample transfer only; no open handling of biohazardous material	
**25. Waste/disposal requirements**	Standard biohazardous waste disposal or incineration of consumables, no high temperature incineration required	Small environmental footprint; recyclable or compostable plastics for test cartridges and other materials after decontamination, no incineration required

**26. Internal QC - reagents**	Procedural (reagent-addition) control internalized in cartridge for each individual test run; positive and/or negative control for internal QC available for purchase separately	Procedural (specimen-addition/sample adequacy) control internalized in cartridge for each individual test run; positive and/or negative control for internal QC provided in each box of test kits
**27. Device control**	Indicator of instability or expiration	Indicator of instability, expiration, inadequate sample and incorrect procedure and/or use but not as an additional component
**28. Patient identification capability**	Yes – simple, self-contained way to indicate a patient identifier	
**29. Result display and interpretation**	Result can be read with the naked eye with minimal instructions for interpretation required by user, or with an integrated reader (See Appendix 1) that supports enhanced test performance	
**PRICING AND ACCESSIBILITY**		
**30. Target list price[5] per test (excluding the cost of a reader)**	<$3 USD for a low complexity test (e.g. rapid diagnostic test) that meets the minimal intended use and clinical sensitivity and specificity TPP specifications;<br><$12 USD for a moderate/high complexity test (e.g. disposable single use molecular test) that meets the optimal intended use and clinical sensitivity and specificity TPP specifications	
**31. Regulatory requirements**	WHO PQ or other stringent regulatory body (e.g. FDA or CE mark)	

Appendix 1: Requirements for RDT reader (if required)

Adapted from RDT reader TPP prepared by the Murtagh Group, LLC (2014)

READER CHARACTERISTICS (if reader is required)	Minimal	Optimal
**1. Ease of Use**	No more than 3 operator steps (position RDT (cassette/strip) as required by the reader; take image or scan; read result); simple test menu; integrated LCD screen; simple key pad or touchscreen with icons	
**2. Size**	Small, portable table-top or hand-held device; or disposable reader	

[5] List Price– the price the manufacturer has arrived at for the product, taking into account the cost of goods and other factors (e.g., margin); the list price does not include any volume or other discounts or potential markup for distribution or other costs, including freight, taxes, etc. This cost is assumed a volume production and the prices listed in the TPP are considered for public health preferential pricing in low- and middle-income countries only.

**3. Power Requirements**	Standard AA/AAA batteries or rechargeable battery with 8-hour operation between charges. Rechargeable battery lifetime > 2 years ~~and less than $50 USD~~	
**4. Service, Maintenance and Calibration**	Routine preventive maintenance no more than 30 minutes 1x per week (with hands on time <10 minutes). Mean time between failures of at least 36 months or 30,000 tests, whichever occurs first. Self-check alerts operator to reader errors or warnings; and ability to be calibrated remotely, or no calibration needed	
**5. Patient Identification Capability**	Manual entry of alphanumeric patient identifier keypad or touchscreen compatible with protective gloves	Same, plus bar code, RFID or other reader
**6. Result display; result interpretation**	Easy pictorial display: positive, negative, or invalid for each target analyte; no instructions for interpretation required	
**7. Data acquisition and display**	Able to add information (patient ID, operator ID, date, location, etc.); able to store patient results; able to print out results utilizing commoditized paper products (i.e. standard paper specifications and sizes)	
**8. Connectivity**	Reader has integrated global positioning system (GPS) module	If combined with a reader, internally integrated GPS/ general packet radio service (GPRS) module and conformity with HL7 messaging standards
**9. Data export**	Full data export over mobile phone network	Full data export over mobile phone network (data transmission can automatically select between GPRS or more advanced networks and global system for mobile communication (GSM), based on available coverage)<br>GPRS should be able to utilize the internet file transfer protocol to transmit data: data transfer should be initiated every 6–12 hours automatically by the reader; data can be exported in a format compatible with HL7 standards, where appropriate; instrument tracks and transmits quality assurance data over time (e.g. identify shifts or trends)
**10. Regulatory Requirements**	GMP compliant, ISO 13485:2016 certified and authorized for use by a stringent regulatory authority	
**11. Cost of Reader**	Reader cost included in the list price of the test	

Note: Some common characteristics for POCTs were defined using the WHO NG TPP previously developed, located here: http://www.who.int/reproductivehealth/topics/rtis/pocts/en/
