# Supplementary material for: Developing target product profiles for Neisseria gonorrhoeae diagnostics in the context of antimicrobial resistance: An expert consensus
Source: PLoS One. 2020 Sep 1;15(9):e0237424. doi: 10.1371/journal.pone.0237424 (PMC7462286; doi:10.1371/journal.pone.0237424)
Supplement: S2 File — (PDF) [file pone.0237424.s002.pdf]

Target product profile
for a test to identify susceptibility/resistance of gonorrhoea to antibiotics to facilitate antibiotic stewardship

FIND, Campus Biotech. Chemin des Mines 9, 1202 Geneva, Switzerland
T +41 (0)22 710 05 90, F +41 (0)22 710 05 99, www.finddx.org

Target product profile for a test to identify susceptibility/resistance of gonorrhoea to antibiotics to facilitate antibiotic stewardship		
**Characteristic**	**Minimal**	**Optimal**
**SCOPE**		
**1. Intended use**	A test to confirm Neisseria gonorrhoea (NG) infection and to detect genetic markers of antibiotic susceptibility[1]/resistance for NG to facilitate antibiotic stewardship[2]	A rapid disposable test to detect NG only or and NG and Chlamydia trachomatis (CT) infection as previously defined,[3] plus detection of genetic markers of antibiotic susceptibility/resistance for NG to facilitate antibiotic stewardship
**2. Target use setting**	Level 2[4] healthcare facility[5] (e.g. District Hospital and peri-urban clinics) defined as having a functioning laboratory with trained personnel, water, electricity with intermittent surges and/or outages, limited climate control, dust, and medical staff onsite	Primary health care settings including health posts (Level 1[1])
**3. Test format**	Fully integrated instrument[6] designed for use in level 2 facilities in combination with a self-contained, disposable assay cartridge containing all required reagents to execute a test from sample to result	Standalone, non-instrumented, single use, disposable diagnostic test for use in level 1 facilities preferred, reader[7] optional and only if required to achieve the intended use
**4. Target users**	Trained laboratory personnel (e.g., 1-2 year certificates) and any health worker with a similar or superior training level	Minimally skilled healthcare personnel (e.g. 3-6 months, able to operate an integrated test with minimal additional steps)
**5. Training required**	< 90 minutes	30 minutes

[1] Genetic markers of antibiotic susceptibility/resistance refers to genetic resistance mutations, the absence of which are consistent with wild type genotype and antibiotic susceptibility. Detection of the presence or absence of resistance mutations does not provide confirmation of susceptibility, but rather predict the likelihood of resistance, since mechanisms of resistance other than those detected by specific genetic marker(s) may exist.

[2] Antibiotic stewardship refers to determining targeted therapy with antibiotics to preserve the use of second or third line antibiotics

[3] See the target product profile for a rapid, low-cost diagnostic to distinguish gonorrhoea from Chlamydia infection at primary care

[4] Ghani AC, Burgess DH, Reynolds A, Rousseau C (2015). Expanding the role of diagnostic and prognostic tools for infectious diseases in resource-poor settings. *Nature* 528: S50-52

[5] The intended use for the minimal case in level 2 could serve both naïve cases as well as those referred from lower levels of the health system

[6] Instrument specific characteristics are defined in Appendix 1

[7] Reader specific characteristics are defined in Appendix 2

**6. Target analytes**	Detection of genetic markers of susceptibility/resistance[8] to any one of the following antibiotics[9]:<br>• ciprofloxacin<br>• ceftriaxone<br>• cefixime<br>• penicillin<br>• gentamicin<br>• azithromycin<br>• zoliflodacin	Detection of genetic markers of susceptibility/resistance to at least 2 of the following antibiotics:<br>• ciprofloxacin<br>• ceftriaxone<br>• cefixime<br>penicillin<br>• gentamicin<br>• azithromycin<br>• zoliflodacin
**Characteristic**	**Minimal**	**Optimal**
**TEST OPERATIONAL CHARACTERISTICS**		
**7. Test kit**	All materials required for test procedure are integrated on the test cartridge except for consumables required to diagnose one individual, included in packaged, self-contained kit	
**8. Specimen[10]**	Women: self-collected and provider collected high vaginal swabs, urine preferred<br>Men: urethral swab acceptable, urine preferred<br>Same test format able to accept multiple specimen types in order to achieve results for men and women	Women: same as minimal<br>Men: urine, and rectal and pharyngeal swabs<br>Same test format able to accept multiple specimen types in order to achieve results for men and women
**9. Specimen preparation**	Minimal sample processing; no more than one operator step	Integrated, no sample preparation required by user
**10. Ease of use**	No more than three operator steps none of which is timed or labour intensive	One operator step (none of which has a timed interval), excluding waste disposal

[8] Examples include: gyrA locus related to DNA gyrAse for ciprofloxacin, beta-lactamase and mosaic type of penA for penicillin, mosaic-like sequences within the *mtr* (*m*ultiple *t*ransferable *r*esistance) efflux pump locus for azithromycin, and the mosaic type of penA for expanded spectrum cephalosporins (ceftriaxone and cefixime), etc.

[9] There are considerable differences in geographical patterns of resistance between high and low- and middle-income countries; therefore, detection of resistance markets to particular antibiotics should be considered depending on intended markets for use

[10] Sensitivity and specificity for rectal and pharyngeal swabs is not yet determined

**11. Duration of sample stability**	Immediate testing of the sample once collected	
**12. Additional consumables required but not provided within the test kit**	None, except for specimen collection	
**TEST PERFORMANCE CHARACTERISTICS**		
**13. Clinical sensitivity to predict resistance**	≥98% sensitivity for predicting gonococcal resistance to the antibiotics listed in target analytes	>98% sensitivity for predicting gonococcal resistance to the antibiotics listed in target analytes
**14. Clinical specificity to predict resistance**	≥95% specificity for predicting gonococcal resistance to the antibiotics listed in target analytes	>98% specificity for predicting gonococcal resistance to the antibiotics listed in target analytes
**15. Time to result**	≤60 minutes	≤20 minutes
**16. Internal process control**	A full internal process control must be integrated into the assay cartridge and the instrument	
**17. Positive / Negative controls**	External positive and negative controls are not required for each test but are performed daily;<br>Control for sample adequacy is required for self-collected swabs	External positive and negative controls are not required for each test and do not need to be run daily;<br>Control for sample adequacy is required for self-collected swabs
**OPERATIONAL CHARACTERISTICS**		
**18. Operating conditions**	Operation between 15°C and 40°C at an altitude up to 2000 meters<br>Extremely low relative humidity to condensing humidity<br>Result interpretation in low light settings	Same, plus operation between 10°C and 45°C at an altitude up to 3000 meters preferred
**19. Cold chain**	None required at any point	
**20. Test kit stability and storage conditions**	12 months, stable between 2-35ºC, 70% humidity, 3000 meters altitude<br>Indicator of instability or expiration	18 months, stable between 0-50ºC, 90% humidity, 3000 meters altitude<br>Indicator of instability or expiration

**21. Environmental tolerance of packaged test kit**	Transport stress (48 hours with fluctuations up to 45°C and down to 0°C);<br>Tolerate exposures between 2°C and 45°C at an altitude up to 2000 meters, up to and including condensing humidity	Transport stress (48 hours with fluctuations up to 50°C and down to 0°C);<br>Tolerate exposures between 2°C and 45°C at an altitude up to 3000 meters, up to and including condensing humidity
**22. Safety precautions (bio-safety requirements)**	Closed, self-contained system; unprocessed sample transfer only; no open handling of biohazardous material	
**23. Waste/disposal requirements**	Standard biohazardous waste disposal or incineration of consumables, no high temperature incineration required	Small environmental footprint; compostable plastics for test cartridges and other materials after decontamination
**24. Result display; result interpretation**	Result lists each antibiotic tested and report whether a resistant marker(s) was detected	Same as minimal and the result can be read with the naked eye with minimal instructions for interpretation required by user,<br>or with an integrated reader if addition of the reader supports enhanced test performance (See Appendix 1 for reader requirements)
**25. Connectivity and data export**	Connectivity required to support surveillance, See Appendix 1 for instrument requirements	Connectivity required to support surveillance, See Appendix 2 for reader requirements
**PRICING AND ACCESSIBILITY**		
**26. Regulatory requirements**	WHO PQ or other stringent regulatory body (e.g. FDA or CE mark)	
**27. Target list price[11] per test (excluding the cost of a reader or instrument)**	< $25 USD at volume production	< $15 USD at volume production

Appendix 1: Instrument Requirements

**INSTRUMENT CHARACTERISTICS (if**	**Minimal**	**Optimal**

[11] List Price– the price the manufacturer has arrived at for the product, taking into account the cost of goods and other factors (e.g., margin); the list price does not include any volume or other discounts or potential markup for distribution or other costs, including freight, taxes, etc. This cost is assumed a volume production and the prices listed in the TPP are considered for public health preferential pricing in low and middle income countries only.

instrument is required)		
1. **Size**	Small, table-top instrument (50 cm x 75 cm by 50 cm, or smaller)	
2. **Weight**	≤25 kg	≤10 kg
3. **Power Requirements**	Local 110-220 AC mains power, plus uninterruptable power supply (UPS) to complete current cycle. includes rechargeable battery back-up (8-hour operation). External UPS and circuit protector included with the system	Same, plus UPS and circuit protector must be integrated within the system
4. **Throughput**	Throughput of 16 or more sample runs per instrument per 8-hour day	
5. **Service, Maintenance and Calibration**	Routine preventive maintenance no more than 30 minutes 1x per week (with hands on time <10 minutes). Mean time between failures of at least 36 months or 30,000 tests, whichever occurs first. Self-check alerts operator to instrument errors or warnings; and ability to be calibrated remotely, or no calibration needed	
6. **Patient Identification Capability**	Manual entry of alphanumeric patient identifier keypad or touchscreen compatible with protective gloves	Same, plus bar code, RFID or other reader
7. **Result display; result interpretation**	Qualitative result reported	
8. **Data acquisition and display**	On-instrument visual readout with ability to function in various lighting conditions ranging from direct sunlight to low ambient light conditions. Able to add information (patient ID, operator ID, date, location, etc.)	
9. **Connectivity**	• Integrated Local Area Network (LAN) port<br>• Integrated WiFi 802.11b/g/n<br>• USB 3.0<br>• Internally designatable static IP address<br>• Support for DHCP issued IP addresses<br>• Support for HTTPS and SFTP protocols<br>• Integrated global positioning system (GPS)<br>Ability to update connectivity software stack via USB or LAN	Same as minimal, plus:<br>• Multi-band GSM chipset 2G, 3G, LTE<br>• Integrated Bluetooth 5.0<br>• Integrated WiFi 802.11ac<br>Bi-directional communication – ability to update connectivity software stack
10. **Data export**	Export of all instrument and test data over integrated hardware. Secured data export with end-to-end encryption. Data export in .CSV file format. Configurable destination IP and DNS address. User initiated data	Same as minimal, plus scheduled/automatic data export using interoperable standards via the Global System for Mobile Communications SMS.

	export. Connectivity to external printer.	
**11. Regulatory Requirements**	GMP compliant, ISO 13485:2016 certified and authorized for use by a stringent regulatory authority (i.e. FDA or CE mark)	
**12. List price[5] of Instrument**	<$5000 USD at volume production	<$1000 USD at volume production

Appendix 2: Requirements for RDT reader (if required)

Adapted from RDT reader TPP prepared by the Murtagh Group, LLC (2014)

**READER CHARACTERISTICS (if reader is required)**	**Minimal**	**Optimal**
**1. Ease of Use**	No more than 3 operator steps (position RDT (cassette/strip) as required by the reader; take image or scan; read result); simple test menu; integrated LCD screen; simple key pad or touchscreen with icons	
**2. Size**	Small, portable table-top or hand-held device; or disposable reader	
**3. Power Requirements**	Standard AA/AAA batteries or rechargeable battery with 8-hour operation between charges. Rechargeable battery lifetime > 2 years ~~and less than $50 USD~~	
**4. Service, Maintenance and Calibration**	Routine preventive maintenance no more than 30 minutes 1x per week (with hands on time <10 minutes). Mean time between failures of at least 36 months or 30,000 tests, whichever occurs first. Self-check alerts operator to reader errors or warnings; and ability to be calibrated remotely, or no calibration needed	
**5. Patient Identification Capability**	Manual entry of alphanumeric patient identifier keypad or touchscreen compatible with protective gloves	Same, plus bar code, RFID or other reader
**6. Result display; result interpretation**	Easy pictorial display: susceptible, not susceptible, no gonorrhoea detected, or invalid for each assay; no instructions for interpretation required.	
**7. Data acquisition and display**	Able to add information (patient ID, operator ID, date, location, etc.); able to store patient results; able to print out results utilizing commoditized paper products (i.e. standard paper specifications and sizes)	

**8. Connectivity**	Reader has integrated global positioning system (GPS) module	If combined with a reader, internally integrated GPS/ general packet radio service (GPRS) module and conformity with HL7 messaging standards
**9. Data export**	Full data export over mobile phone network	Full data export over mobile phone network (data transmission can automatically select between GPRS or more advanced networks and global system for mobile communication (GSM), based on available coverage)<br>GPRS should be able to utilize the internet file transfer protocol to transmit data: data transfer should be initiated every 6–12 hours automatically by the reader; data can be exported in a format compatible with HL7 standards, where appropriate; instrument tracks and transmits quality assurance data over time (e.g. identify shifts or trends)
**10. Regulatory Requirements**	GMP compliant, ISO 13485:2016 certified and authorized for use by a stringent regulatory authority	
**11. Cost of Reader**	Reader cost included in the list price of the test	
